# Obesity, Gut Microbiota, and Metabolome: From Pathophysiology to Nutritional Interventions

**DOI:** 10.3390/nu15102236

**Published:** 2023-05-09

**Authors:** Zivana Puljiz, Marko Kumric, Josip Vrdoljak, Dinko Martinovic, Tina Ticinovic Kurir, Marin Ozren Krnic, Hrvoje Urlic, Zeljko Puljiz, Jurica Zucko, Petra Dumanic, Ivana Mikolasevic, Josko Bozic

**Affiliations:** 1 Laboratory for Bioinformatics, Faculty of Food Technology and Biotechnology, University of Zagreb, 10000 Zagreb, Croatiajzucko@pbf.hr (J.Z.); 2 Department of Pathophysiology, University of Split School of Medicine, 21000 Split, Croatia; marko.kumric@mefst.hr (M.K.); dinko.martinovic@mefst.hr (D.M.); tticinov@mefst.hr (T.T.K.);; 3 Department of Endocrinology, Diabetes and Metabolic Diseases, University Hospital of Split, 21000 Split, Croatia; 4 Department of Internal Medicine, University of Split School of Medicine, 21000 Split, Croatia; 5 Department of Gastroenterology and Hepatology, University Hospital of Split, 21000 Split, Croatia; 6 Medical Laboratory Diagnostic Division, University Hospital of Split, 21000 Split, Croatia; 7 Department of Gastroenterology and Hepatology, University Hospital Centre Rijeka, School of Medicine, University of Rijeka, 51000 Rijeka, Croatia

**Keywords:** obesity, gut microbiota, metabolome, Mediterranean diet, Roux-en-Y gastric bypass, ketogenic diet

## Abstract

Obesity is a disorder identified by an inappropriate increase in weight in relation to height and is considered by many international health institutions to be a major pandemic of the 21st century. The gut microbial ecosystem impacts obesity in multiple ways that yield downstream metabolic consequences, such as affecting systemic inflammation, immune response, and energy harvest, but also the gut–host interface. Metabolomics, a systematized study of low-molecular-weight molecules that take part in metabolic pathways, represents a serviceable method for elucidation of the crosstalk between hosts’ metabolism and gut microbiota. In the present review, we confer about clinical and preclinical studies exploring the association of obesity and related metabolic disorders with various gut microbiome profiles, and the effects of several dietary interventions on gut microbiome composition and the metabolome. It is well established that various nutritional interventions may serve as an efficient therapeutic approach to support weight loss in obese individuals, yet no agreement exists in regard to the most effective dietary protocol, both in the short and long term. However, metabolite profiling and the gut microbiota composition might represent an opportunity to methodically establish predictors for obesity control that are relatively simple to measure in comparison to traditional approaches, and it may also present a tool to determine the optimal nutritional intervention to ameliorate obesity in an individual. Nevertheless, a lack of adequately powered randomized trials impedes the application of observations to clinical practice.

## 1. Introduction

Obesity is a disorder identified by an inappropriate increase in weight in relation to height, mainly owing to fat tissue accumulation, and is widely considered a pandemic of the 21st century. The available data indicate that the etiological triad of genome, diet, and gut microbiota plays a crucial role in the pathophysiology of obesity. The gut microbial ecosystem impacts obesity in multiple ways that yield downstream metabolic consequences, such as affecting systemic inflammation, immune response, and energy harvest, but also gut–host interface (gastrointestinal mucus layer, epithelial permeability, and inflammation of the digestive system) [1]. The gut microbiota constitution can govern the efficacy of energy harvest from foodstuffs, and changes in dietary style have been related to changes in the composition of gut microbial populations. Our ability to examine the microbiota constitution was significantly improved with the advent of a metagenomic approach, which has already granted the production of the human gut microbiome gene catalogue and enabled stratification of individuals according to their respective gut genomic profile into different enterotypes [2,3,4]. Furthermore, metabolomics, a systematized study of low-molecular-weight molecules that take part in metabolic pathways, represents a serviceable method for elucidation of the crosstalk between hosts’ metabolism and gut microbiota.

Metabolomics is a promising tool to upgrade current clinical assessments based on single metabolites by identifying metabolic signatures (biomarkers) that represent global biochemical changes in disease and predict responses to side effects of drug treatment (pharmacometabolomics) [5]. The gut microbiota composition can be affected by various external factors, with dietary habits being recognized as the most important one. In many pathological states, such as celiac disease, irritable bowel syndrome (IBS), and also some neurological disorders (epilepsy), specific dietary regimens such as a Mediterranean diet, ketogenic diet, and gluten-free diet are recognized as therapeutic. It seems that all of the above noted dietary patterns can affect the gut microbiota composition, especially when implemented over longer time periods [6]. Accordingly, it is worth mentioning that nutritional intervention may serve as an efficient therapeutic approach to support weight loss in obese individuals, yet no agreement exists in regard to the most effective dietary protocol, both in the short and long term [6].

In the present review, we provide an overview of the current evidence concerning the association between gut microbiota and obesity. As identification of the gut microbiota–metabolites is warranted in order to clarify the interactions between the host and the intestinal flora, we investigated changes in the metabolome, which is a necessary step to appropriately model metabolic interactions inside the intestinal ecosystem. Hence, in this review, we primarily discussed clinical and preclinical studies that explored the association of obesity and related metabolic disorders with various gut microbiome profiles, and the effects of weight loss interventions with different dietary approaches on gut microbiome and the metabolome.

## 2. Gut Microbiota and Obesity

Gut microbiota represents one of the most complex ecosystems in nature, harboring large bacterial populations in the intestine and colon, most of which are anaerobic bacteria (95% of total organisms) [7]. Gut microbiota in humans is mainly composed of five distinct phyla: *Actinobacteria*, *Bacteroidetes*, *Verrucomicrobia*, *Firmicutes*, and *Proteobacteria*, with *Bacteroidetes* and *Firmicutes* representing as much as 90% of all gut bacteria [8,9,10,11]. Nevertheless, a large between-subjects diversity in gut microbiome is present [9]. Three bacterial clusters have been described so far: *Bacteroides* (enterotype 1), *Prevotella* (enterotype 2), and *Ruminococcus* (enterotype 3) [8]. The *Prevotella* enterotype is related to a high-carbohydrate diet, whereas the *Bacteroides* enterotype has been mainly correlated with a diet rich in animal fat and protein [9]. This simplified classification, although not generally accepted [12], may contribute to a more comprehensive understanding of the complex relations operating between gut microbiota and obesity.

Dysbiosis, a disturbance in microbiota composition, has been linked to three distinct mechanisms that can all take place at the same time: (1) the loss of “beneficial” bacteria, (2) an excessive growth of potentially hazardous bacteria, and (3) a reduction in the microbial diversity [13,14]. A growing amount of evidence suggests that changed microbiota can have an impact on host physiology through a number of different channels, including improved energy harvest, changes in the immune system, metabolic signaling, and inflammatory pathways [15,16,17]. For instance, evidence suggests that microbiota may be a significant contributor that favors weight gain, fat storage, and insulin resistance [18]. The gut bacteria are involved in energy homeostasis via formation of short chain fatty acid (SCFAs) and extraction of energy through fermentation processes [19]. The gut microbiota may also enable increased intestinal absorption by increasing villous vascularization and increased triglyceride storage via modulation of fasting-induced adipose factor, a lipoprotein lipase (LPL) activity inhibitor [20]. Interestingly, a study of a mice model demonstrated that the obese phenotype may be transmissible by transplanting the gut microbiota [21]. Subsequently, a focus was placed on the identification of bacterial strains that are implicated in the pathophysiology of obesity.

In general, obese individuals are characterized by decreased bacterial diversity [22,23,24], as well as gene richness [25,26]. In fact, recent studies in European populations have demonstrated that people with less complex microbiomes have greater obesity in general, higher levels of inflammatory markers, more pronounced insulin resistance, and dyslipidemia [27]. Likewise, obese and overweight individuals with low diversity microbiota demonstrate an increase in microbial richness upon the introduction of an energy-restricted diet [28]. Unlike the original reports suggested that obesity is associated with a higher ratio of *Firmicutes*-to-*Bacteroidetes* [29], more recent studies failed to demonstrate such an association, suggesting that the ratio is not a significant factor in human obesity [30,31,32,33,34,35]. In fact, the mere fact that most studies showed decreased bacterial diversity in obesity is also suggestive that separate changes in gut microbiome composition at family, genus, or species level are more relevant for pathophysiology of obesity than the aforementioned ratio. Nonetheless, many researchers have studied ways in which diet can modulate the *Firmicutes*-to-*Bacteroidetes* ratio. In accordance with the previously noted findings, early studies demonstrated that the *Firmicutes*-to-*Bacteroidetes* ratio is increased in obese individuals but decreases upon weight loss after bariatric surgery and/or calorie-restriction diets, whereas other studies mostly failed to demonstrate the same [36,37]. It is however important to note that all these studies were largely underpowered to demonstrate any difference and, thus, perhaps the strongest evidence implying a lack of link between the *Firmicutes*-to-*Bacteroidetes* ratio and obesity came from a meta-analysis that showed no difference in the abundance of *Firmicutes* and *Bacteroidetes*, nor the *Firmicutes*-to-*Bacteroidetes* ratio [38]. Overall, although the mere ratio of these two phyla is obviously not a representative indicator of obesity, the studies that covered this topic revealed some important findings with regard to the microbiota–obesity interconnection. Specifically, it challenged the concept of a unique taxonomic signature related to obesity, thus steering future research into identifying taxonomic markers for stratification of patients into subgroups.

Apart from *Bacteroidetes* and *Firmicutes*, other studies have associated obesity with distinct bacteria including family *Christensenellaceae* and the genera *Akkermansia*, *Bifidobacteria*, *Methanobacteriales*, and *Lactobacillus* [39]. Specifically, the *Christensenellaceae* family was recently associated with weight loss and several gene expression pathways in subcutaneous adipose tissue, such as protein–amino acid N-glycation, whereas its relative quantity was inversely related to the host BMI [39,40]. Moreover, supplementation with *A. muciniphila* was shown to improve metabolic parameters in obese patients [41]. Furthermore, unlike the abundance of *Lactobacillus reuteri* and *L. gasseri* that positively correlated with obesity, *L*. *paracasei* negatively correlated with obesity, indicating that obesity-related bacteria are species specific, and that bacteria in the same genus may yield opposing effects [42]. The role of bacteria in obesity seems to be strain specific, i.e., both beneficial and harmful bacteria can exist within the same taxon. Hence, it is rather challenging to categorize obesity-related bacterial communities according to their taxonomic relationships.

The relationship between obesity and gut microbiota is a two-way street. Namely, gut microbiota may promote obesity through the direct interaction between the microbiota and cells in the GI tract or indirect interaction between produced metabolites and remote organs. The underlying mechanisms may include the effects of gut microbiota on fat storage, appetite, absorption of energy, circadian rhythm, and chronic inflammation, all of which can promote obesity [43]. Finally, results from interventions that affect gut microbiota (fecal microbiota transplant, use of pre-, pro-, and synbiotics) have pointed out that such changes might herald favorable outcomes in obese individuals [44].

A very important, yet commonly neglected, aspect concerning the research of gut microbiome are technical challenges in defining the gut microbiome composition. This issue is well illustrated in a meta-analysis by Walters et al. [45]. Namely, the results of their meta-analysis, in which only studies involving high-throughput sequencing of the 16S rRNA gene were analyzed, showed that the gut microbiota composition is not clustered by subject’s body mass index (BMI) but rather by study, suggesting that the per-study effect exceeds the biological effect. Moreover, additional bias can arise as a consequence of various differences in data analysis and sample processing, such as the method of DNA extraction, the method of sequencing, the choice of primers, and the bioinformatic analysis (taxonomy database and assignment algorithm used) [46,47,48].

## 3. Metabolites and Obesity: The Role of Gut Microbiota

Although obesity is an established metabolic disorder, the pathophysiological mechanisms linking weight gain with metabolic profiles are still elusive. Gut microbiome dysbiosis seen in obese individuals may have an impact on the metabolism and excretion of microbiota byproducts, and may therefore affect hosts’ physiology. Recent data imply that the intestinal microbiota of obese individuals is characterized by alterations in any circulating metabolites and is associated with fasting levels of a number of metabolites, such as lipids and lipid-like metabolites, amino acids and their byproducts, bile acids derivatives, and metabolites derived from the degradation of polyphenols, choline, carnitine, and purines [49,50]. Metabolite profiling might represent an opportunity to methodically establish predictors for obesity control that are relatively simple to measure in comparison to traditional approaches. In addition, these predictors may also constitute a tool to evaluate responses/effects to different intervention approaches.

A meta-analysis by Moore et al. recognized 37 metabolites that are associated with BMI, identifying 18 of them, including butyrylcarnitine, a marker of whole-body fatty acid oxidation, and histidine for the first time [51]. The meta-analysis was performed on studies that included both American and Chinese populations and it included 947 patients. Perhaps the biggest drawback of this meta-analysis was the fact that it included cross-sectional studies exclusively. On the other hand, Zhao et al. performed a 5-year follow-up cohort study that included 300 Mexican American healthy women [52]. At baseline, the authors observed 7 metabolites that are associated with BMI in both cohorts. The metabolites were as follows: asparagine, glycine, glutamic acid, kynurenic acid, methyl succinate, urate, and serine. At the end of the follow-up period, the investigators identified 6 metabolites (acetylcholine, acetyl glycine, hippuric acid, leucine, urate, and xanthine) whose baseline levels heralded significant weight gain during a 5-year follow-up in both cohorts. It is worth mentioning that 4 of these were the same as metabolites from the aforementioned meta-analysis. The only metabolite for which an association with BMI had not been established previous to Zhao et al.’s study was methylsuccinate, a metabolite involved in isoleucine catabolism [53].

Accordingly, Geidenstam et al. showed that metabolite risk score may represent a useful tool on both the individual and population levels, and markedly so if additional metabolites are recognized to improve predictive power [54]. It is important to address that none of the anthropometric, lifestyle, and glycemic risk factors that are standardly measured may fully explain the association between changes in BMI (ΔBMI) and metabolite risk score. The proportion of ΔBMI variance explained by the metabolite risk score was similar to that for the best predictive risk factors (glycemic measures). The above-noted metabolite risk score is comprised of eight metabolites, including malate, niacinamide, xanthine, tyrosine, uridine, and three lipids (TAG 56:6, SM24:0, and TAG 56:2), and could point to independent processes related to weight gain. Regarding the lipids, Geidenstam et al. showed that TAG 56:6 positively correlates with ΔBMI, whereas TAG 56:2 and SM 24:0 show the opposite trend. Such a pattern is in corroboration with previous findings showing that lipids with varying saturation levels have the opposite connection with obesity-related phenotypes [54]. Furthermore, the authors observed that SM 24:0 and TAG 56:2 are associated with decreased weight gain in both of the analyzed cohorts (Mexico City Diabetes Study and Framingham Heart Study), and increased diabetes risk in the former cohort (i.e., they have negative effect sizes in the metabolite risk score that is protective against diabetes), while TAG 56:6 had the opposite direction of effect. Overall, these results suggest that metabolite risk score can be employed as an independent predictor for studying weight gain.

Studies that have examined branched chain amino acid (BCAA) supplementation in both animal and human models suggest that circulating AAs can contribute to insulin resistance, conceivably by affecting insulin signaling in skeletal muscles [55,56]. On a cellular level, the underlying pathophysiological mechanisms seem to include activation of the mTOR, JUN, and IRS1 signaling pathways [57,58]. On the other hand, several authors have shown an improvement of glucose homeostasis in animals fed with a leucine-rich diet [59]. In line with this, it is noteworthy to mention that multiple amino acids, particularly BCAAs, are modulators of insulin secretion [60]. In particular, from a panel of more than 60 metabolites, aromatic amino acids and BCAAs appear to be the best predictors for the future development of diabetes mellitus. A single measurement of these amino acids in fasting state has demonstrated incremental benefit over standard risk factors (such as fasting glucose, dietary patterns, and BMI) [61]. Obese adults, with or without diabetes mellitus, were shown to have hyperaminoacidemia. Specifically, increases in the concentrations of the BCAAs, alongside phenylalanine and tyrosine, have been described [62]. Dissimilar to other essential amino acids, BCAAs are degraded in skeletal muscle, and it has been shown that BCAA circulating levels are elevated post-prandially [63]. Such elevations in BCAAs may influence glucose homeostasis since oxidation of BCAAs spares glucose utilization in skeletal muscle [64]. McCormack et al. demonstrated that an increase in circulating BCAAs is significantly associated with obesity in both children and adolescent populations and is capable of predicting the development of insulin resistance [65]. In fact, increased concentrations of BCAAs are also present in young obese individuals, and their metabolomic profiles are in accordance with increased catabolism of BCAAs, whereas elevations in BCAAs are positively associated with insulin resistance measured after 18 months, independent of the initial BMI [65]. The background of obesity-related increases in the concentrations of BCAAs remains elusive. It is plausible that obese people have more BCAAs in their diet, which could in the context of a high-fat diet characteristic of diet-induced obesity also portend to insulin resistance development [66]. Conversely, insulin resistance may in turn lead to the failure of insulin’s physiologic capacity to suppress BCAA levels [67]. Shaham et al. have shown that some obese individuals display differential sensitivity to insulin-induced suppression of proteolysis compared to lipolysis by using metabolite profiling technology [68]. Interestingly, they showed that elevated BCAA concentration in serum may appear long before other indices of insulin resistance become abnormal [69]. In a cross-sectional study that included 182 non-diabetic individuals, Seibert et al. performed a measurement of 24 plasma amino acids and an insulin suppression test. The study showed that in 14/24 amino acids, concentrations were significantly higher in men than women, whereas glycine was found to be lower in men. The majority of these amino acids positively correlated with steady-state plasma glucose (SSPG), while glycine concentration demonstrated a negative correlation. Leucine, isoleucine, glutamic acid, and tyrosine concentrations showed the strongest correlation with SSPG. In comparison to women, men were more prone to demonstrate a rather unfavorable amino acid profile, with a higher concentration of amino acids associated with higher insulin resistance and less glycine [70].

In recent years, a possible connection between serum AAs, gut microbiome, and obesity was explored. In a pivotal study, Calvani et al. demonstrated a plausible link between gut microflora metabolism and obesity phenotype using a nuclear magnetic resonance (NMR)-based metabolomic approach [71]. The study included 15 morbidly obese male patients (having BMI > 40 kg/m^2^) and 10 heathy controls. The analysis of NMR spectra of urine samples showed a clear distinction between morbidly obese subjects and lean counterparts in this regard. The main metabolites that were involved in discrimination between the two were xanthine, hippuric acid, trigonelline, and 2-hydroxyisobutyrate. All the aforementioned metabolites were higher in lean subjects, with the exception of 2-hydroxyisobutyrate, which was more abundant in morbidly obese subjects. A very important concept that had also arisen from the findings of this study is the change in urinary metabolic phenotype following bariatric surgery. Namely, hippuric acid and trigonelline levels significantly increased following surgery, whereas 2-hydroxyisobutyrate and xanthine concentrations approximated to the lean subject values. Multiple studies concerning this problem were conducted henceforth, yet most of them were also conducted on relatively small samples, thus failing to reach final conclusions. Nevertheless, some larger studies are worth mentioning in this regard. Liu et al. demonstrated that *Bacteroides thetaiotaomicron*, a glutamate-fermenting commensal, is less abundant in obese individuals in comparison to lean counterparts, and that the amount inversely correlates with serum glutamate concentration [72]. Moreover, it was shown that bariatric surgery led to reversal of these alterations, with regards to both *B. thetaiotaomicron* and glutamate serum levels. On the other hand, gavage with *B. thetaiotaomicron* led to a reduction in serum glutamate concentration, and attenuated diet-induced body-weight gain in a mice model. Multiple important conclusions were reached in an extensive analysis by Org et al., in which the association between gut microbiota and the metabolome was explored using 16S ribosomal RNA gene sequencing and NMR spectroscopy in 531 Finnish adult male subjects [73]. Firstly, a number of connections were established between the gut microbiota composition and serum levels of amino acids, lipids, glucose, and fatty acids. It is important to note that the association existed both with regards to the diversity and richness of microbiota. For instance, acetate levels correlated with microbial diversity, and both acetate and glutamine positively correlated with microbial richness. In addition, associations between multiple bacterial genera and serum concentrations of the above-noted molecules were also established. Furthermore, significant associations between trimethylamine N-oxide (TMAO), a gut microbiota-dependent metabolite, and both coronary artery disease and stroke were found. Finally, the altered composition of microbiota and a significant microbiota–metabolite relationship dependent on glucose tolerance and BMI were detected.

## 4. Effect of Nutritional Interventions on Gut Microbiota and Metabolites

**Figure 1:**
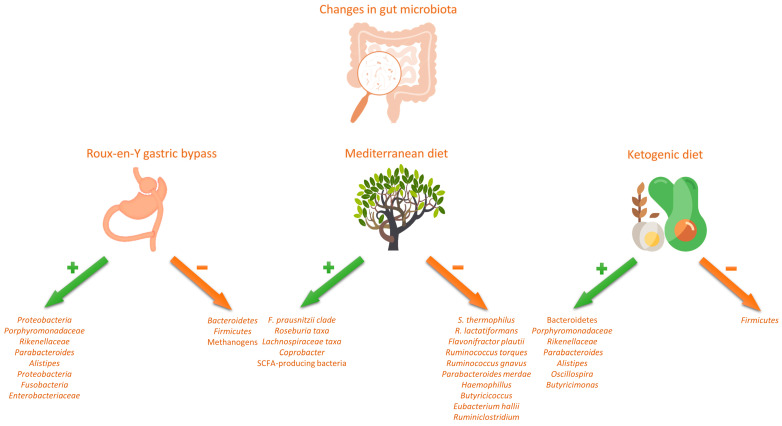
Changes in gut microbiota following dietary interventions (Roux-en-Y gastric bypass, Mediterranean diet, ketogenic diet).

**Figure 2:**
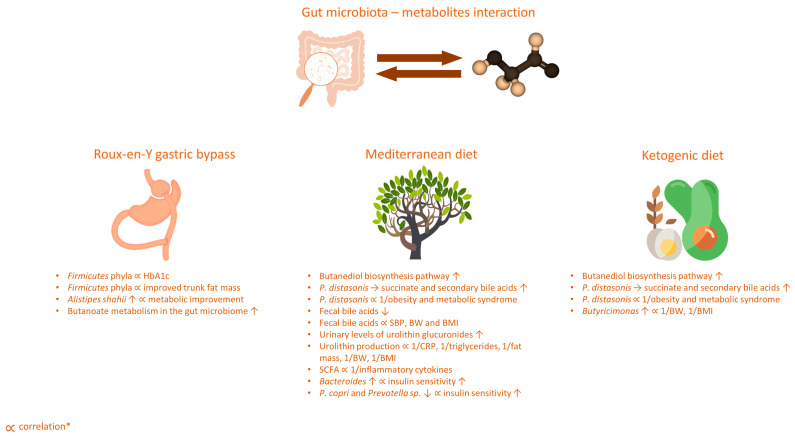
Metabolic changes following dietary interventions (Roux-en-Y gastric bypass, Mediterranean diet, ketogenic diet). * negative correlation was represented as “∝ 1/parameter”. Arrows indicate increase or decrease in bacteria abundance. Abbreviations: BMI: body mass index; BW: body weight; CRP: C-reactive protein; HbA1c: hemoglobin A1c; SBP: systolic blood pressure.

An important note to consider when discussing the effect of nutritional interventions on gut microbiota is that gut microbiome can respond very rapidly to dietary changes, in a way that even short-term (days) animal or plant-based diets may alter microbial community structure and overwhelm inter-individual differences in microbial gene expression. In a seminal study, David et al. demonstrated that changes in gut microbiome are much more prominent with animal-based than plant-based diets. In fact, it is even hypothesized that there is an evolutionary basis that might explain such a rapid switch between carnivorous and herbivorous functional profiles of gut microbiome [74]. The authors suggested that the intake of meat in ancestral humans was relatively volatile, and largely depended on season and hunting success, whereas more abundant plant food offered a sort of a fallback source for nutrients [75]. Therefore, it seems that evolution enabled gut microbiota to rapidly adjust to the imposed changes in diet and subsequently enhance dietary flexibility in humans. On the other hand, dietary interventions seem to elicit transient changes in microbiota composition and it remains elusive as to what duration is warranted to induce a permanent change to the core microbial profile [76]. Finally, it is worth noting that according to recent reports, the composition of one’s gut microbiome is more closely related to specific dietary preferences rather than the nutritional interventions, and, thus, these factors might make it more difficult to see the overall microbiome responses to different diets [77]. An overview of the effects of dietary interventions on gut microbiota and the metabolome is summarized in Figure 1 and Figure 2.

### 4.1. Ketogenic Diet

A ketogenic diet (KD) represents a dietary intervention based on a very low-carbohydrate, high-fat diet which was disputed for a long time. Multiple studies aimed to elucidate the effect of a KD on the composition of gut microbiome in the setting of various pathological states, especially various neurological disorders in which a KD is often recommended by physicians [78].

Preclinical studies consistently demonstrate that this dietary pattern reduces the microbial diversity of the gut, most likely as a result of decreased polysaccharide levels leading to a reduction in bacteria that utilize energy from the gut [79,80,81]. For instance, Olson et al. demonstrated such a reduction in mouse models of epilepsy, but also found that the relative abundance of *Akkermansia muciniphila* and *Parabacteroides* increased [81]. Moreover, the authors even recognized these bacteria as mediators of seizure protection, as it was demonstrated in their study that a KD reduces seizure incidence, an effect abrogated by antibiotic treatment. Notably, Olson et al. also found a link between reduced seizure susceptibility and glutamate in hippocampus, thus further elucidating the role of the gut–brain axis in this regard. Newell et al. reported somewhat different results in a mice model of autism spectrum disorder; although the results were consistent in terms of gut bacteria total abundance, the authors also reported a reduction in the abundance of *A. muciniphila* [80]. Ma et al. also reported interesting preclinical results in which they showed that shaping of gut microbiota by a KD may mitigate the risk of neurodegeneration and offer benefits to neurovascular health [79]. Specifically, aside from decreased diversity, an increased abundance was observed for multiple beneficial bacteria, including the above-noted *A. muciniphila* but also *Lactobacillus* and SCFA-producing bacteria, whereas bacterial taxa that mediate a pro-inflammatory response (*Desulfovibrio* and *Turicibacter*) were down-regulated. Finally, a recent study used metabolomics to elucidate the molecular effects of a KD in models of healthy and tumor xenograft mice models [82]. Metabolomic profiling on plasma samples revealed distinct metabolic fingerprints in the group of mice bearing breast cancer and, importantly, such fingerprints have dissipated following a KD, which resulted in recovery to the metabolic status of healthy mice. Thus, the authors concluded that a KD has a significant molecular effect on tumor growth inhibition beyond a mere constraint of energy supply to tumor cells.

On the other hand, a KD was extensively studied in the setting of neurological disorders. A study in children with refractory epilepsy showed that 6 months of a KD led to a reduction in gut microbiota diversity, but, more importantly, that changes in gut microbiota could predict the efficacy of a KD in terms of a reduction in seizure occurrence. Additionally, a KD caused an increase in *Bacteroidetes* abundance and decrease in *Firmicutes* abundance, which can be interpreted in light of their ratio that we previously discussed [83]. Furthermore, it was demonstrated in multiple sclerosis patients that a KD caused a reduction in terms of both abundance and diversity of gut bacteria at first, yet that a prolonged KD not only restores bacterial diversity, but also leads to more abundant gut microbiota in comparison to the baseline [84]. Notably, it was shown at baseline that the biofermentative function of the colon is markedly impaired in patients with multiple sclerosis. Overall, although the data imply that a KD offers beneficial effects on neurological disorders, at least in part via mechanisms related to gut microbiota, it has to be addressed that adequately powered randomized controlled trials (RCTs) are needed to confirm these notions, given that most data in this regard are based on small-scale studies.

Several studies aimed to elucidate the KD effects on gut microbiota and metabolic pathways in the setting of obesity. Gutiérrez-Repiso et al. compared the effect of three dietary interventions on the gut microbiota profile: KD, Mediterranean diet, and bariatric surgery [85]. In general, perhaps the most important conclusion of the study was the fact that changes in the gut microbiota profile associated with weight loss interventions are not uniform, albeit there was an overlap with regards to certain changes. Specifically, *Porphyromonadaceae* and *Rikenellaceae* families increased following both a KD and bariatric surgery. For both the aforementioned bacteria, a negative correlation with BMI was previously demonstrated, and studies on animal models associated these bacteria with weight loss using probiotics [86,87]. On the genus level, a KD led to an increase in *Parabacteroides*, and *Alistipes*, both of which were negatively associated with BMI in both adult and young populations [88,89,90]. In addition, *Alistipes* was previously shown to predict weight loss following dietary interventions [18]. On the other hand, the amount of *Lactobacillus* was decreased, which is in contrast to the above-noted preclinical studies. At species level, the most relevant finding was an increase in *Parabacteroides distasonis*, bacteria that negatively correlated with obesity and metabolic syndrome in previous studies, and for which a beneficial effect in reducing weight gain, hyperglycemia, and hepatic steatosis was demonstrated [91,92]. More precisely, it is presumed that *P. distasonis* improved metabolic dysfunction via production of succinate and secondary bile acids [92]. Finally, the authors applied PICRUSt to predict metagenome functional content, but found no statistically significant change in functional gut microbiota pathways that was common to the three interventions. Nevertheless, in a KD, most of the changes were on the pathways related to biosynthesis and/or degradation/utilization/assimilation, thus indicating a change in their metabolism. The butanediol biosynthesis pathway, a pathway previously implicated in body weight reduction, thermogenesis, and appetite suppression, was shown to be enriched in both KD and Mediterranean diets [93,94,95,96]. A randomized controlled pilot study explored the effects of a combined KD and synbiotics on gut microbiota [97]. The study showed that the addition of synbiotics does not provide an additional effect on the improvement of microbial diversity observed after 4 months of KD. Specifically, the genus *Oscillospira*, which was shown to have protective associations with visceral fat mass and increased energy expenditure, and *Butyricimonas*, a butyrate-producing bacterium, increased following the intervention [98,99,100]. Furthermore, *Butyricimonas* was positively associated with a greater weight loss and BMI reduction. Despite the addition of synbiotic supplementation not affecting microbial diversity, it seems that its addition may ameliorate inflammation in a process mediated by the gut microbiota alteration.

### 4.2. Mediterranean Diet

The Mediterranean diet (MD) is a dietary pattern that has its roots in the olive-growing regions of the Mediterranean Basin [101]. A high consumption of olive oil, vegetables, fruits, unprocessed cereals, nuts, legumes, fish, and other seafood, moderate consumption of old cheese and red wine, and limited consumption of dairy products and meat are its principal characteristics. Apart from demonstrating benefit in many chronic diseases, a MD’s effects on individuals with obesity is well established, particularly in regard to the gut microbiota composition and the metabolome [102,103,104]. Moreover, since a substantial number of RCTs concerning the interrelation between a MD and gut microbiota were performed (unlike with other dietary interventions), inferences about this dietary regime are perhaps the most reliable ones.

Meslier et al. conducted an RCT which aimed to elucidate how isocaloric MD intervention affected metabolic health, gut microbiome, and the systemic metabolome over the course of 8 weeks [105]. Following the intervention, *Streptococcus thermophilus*, *Ruthenibacterium lactatiformans*, *Flavonifractor plautii*, *Ruminococcus torques*, *Ruminococcus gnavus*, and *Parabacteroides merdae* were significantly reduced, whereas five members of the *Faecalibacterium prausnitzii* clade, alongside several members of the *Roseburia* and *Lachnospiraceae* taxa, were more abundant in the treatment group compared with the control group. The effect of diet was confirmed using integration of the three meta-omics datasets, based on which the authors established a separation of the MD and control group with respect to microbiome diversity, functional modules, and metabolomic profiles. Interestingly, urinary levels of urolithin glucuronides increased following the introduction of the MD, and such changes were concordant with levels of the urolithin producers in the microbiome. Moreover, a negative correlation was established between urolithin production and CRP, triglycerides, fat mass, body weight, and BMI, thus providing a direct link between gut microbiota and functional metabolic alterations resulting from this dietary intervention. Fecal bile acids were reduced in the stools of participants in the MD group, and this may be of relevance as fecal bile acids demonstrated a positive correlation with systolic blood pressure, body weight, and BMI. Finally, the authors concluded that a MD might be helpful in improving insulin sensitivity in individuals with higher levels of multiple *Bacteroides* species, and lower levels of *P. copri* and *Prevotella sp*.

In a separate RCT, the effects of a MD on gut microbiota in obese population over the course of the year were explored [106]. The analysis showed that *Butyricicoccus*, *Eubacterium hallii*, *Haemophillus*, and *Ruminiclostridium* were reduced, whereas *Coprobacter* and uncultured bacterium increased in the energy-restricted MD group in comparison to non-energy-restricted MD. Furthermore, metabolic pathways responsible for the biosynthesis of amino acids, nucleosides, nucleotides, and carbohydrates were significantly different between the two groups of interest. As selected SCFA-producing bacteria were increasingly abundant in both subgroups, and as their number positively correlated with the MD adherence score (MedScore), the authors concluded that MD-induced changes may be modulated by SCFA-producing bacteria regardless of energy restriction. Accordingly, Pagliai et al. explored the effects of a MD on gut microbiota and SCFA production in an RCT with a 3-month follow-up [107]. Although the study suggested that 3 months of MD does not induce major changes in the gut microbiota, the observed changes in SCFA production support the role of a MD in modulating the inflammatory response. Specifically, a negative correlation was observed between SCFA and multiple inflammatory cytokines. Furthermore, the effects of a modified Mediterranean–ketogenic diet (MMKD) on gut microbiota and SCFAs have also been explored in populations with neurological disorders. It was demonstrated in a randomized crossover study that specific gut microbial signatures may herald mild cognitive impairment in Alzheimer’s disease (AD) and that the MMKD may modulate both gut microbiome and metabolites associated with biomarkers of AD in cerebrospinal fluid [108]. Their results were also corroborated by two recent studies that explored the effects of an MMKD on AD and multiple sclerosis [109,110]. Finally, Barber et al. showed in a short-term (2 weeks) MD intervention that despite the relatively small difference in microbiota composition between subjects on a MD and subjects receiving a standard Western-type diet, microbial metabolism was disparate between the groups, as evidenced by urinary metabolite profiles and an abundance of microbial metabolic pathways [111]. Therefore, one can conclude that a sustained MD is needed to elicit relevant gut microbiota changes, whereas even a short-term MD might offer benefit in regard to the metabolome.

### 4.3. Bariatric Surgery

Often regarded as the last resort of morbid obesity treatment, bariatric surgery still represents an important weight-reduction method.

We previously noted that Gutiérrez-Repiso et al. demonstrated the expansion of *Porphyromonadaceae* and *Rikenellaceae* families, *Parabacteroides* and *Alistipes* generum, and *P. distasonis* bacterial species following Roux-en-Y gastric bypass (RYGB) surgery. These results imply that the effector arm of RYGB-induced metabolic alterations may lie in gut microbiota changes. Furthermore, RYGB also led to downregulation of most of the biosynthesis pathways, perhaps as a result of extreme caloric restriction. Pentose phosphate and sugar biosynthesis pathways were also enriched following RYGB, whereas a decrease in the nucleic acid processing pathway was demonstrated. The aforementioned pentose phosphate pathway has been related to obesity, even though the data are rather conflicting [112,113]. By applying a parallel and integrated metagenomic and metabonomic approach, Li et al. demonstrated in a rat model that RYGB surgery induces marked alterations of the main gut bacteria, increasing the concentrations of *Proteobacteria* (as much as 52-fold) while lowering the concentrations of *Bacteroidetes* and *Firmicutes* [114]. Moreover, the authors also aimed to explain their results from a mechanistic standpoint by showing that surgically induced changes in the GI anatomy, flow of nutrients, and weight are also associated with changes in urinary and fecal profiles, thus reflecting increased oligosaccharide fermentation in GI and amine generation, as well as biogenesis of p-cresol and related compounds. Furthermore, a human study further confirmed that RYGB causes profound changes in the gut microbiota composition. Specifically, a marked increase was observed in the *Gammaproteobacteria* class (especially *Enterobacteriaceae*), decrease in *Firmicutes*, and loss of methanogens. The authors argue that shortened small intestinal length, changes in acid exposure to the gastric remnant, and oxygen availability in the colon favored the growth of facultative anaerobes such as *Gammaproteobacteria* over obligate anaerobes such as *Firmicutes*. In addition, it was hypothesized that surgery itself affects food ingestion and digestion, but that it also portends more rapid transit of ingested materials to the colon. Sánchez-Alcoholado et al. compared the effects of two distinct types of bariatric surgeries on gut microbiota and metabolism: RYGB and laparoscopic sleeve gastrectomy (LSG) [115]. Overall, the authors concluded that RYGB yields overall greater gut microbiota differences than SG. Following RYGB, an increase in *Proteobacteria*, *Fusobacteria*, and *Enterobacteriaceae* was observed, whereas *Verrucomicrobiaceae* showed higher levels in SG. Moreover, an inverse correlation was established between *Enterobacteriaceae* and *Veillonella* with cholesterol levels, and a positive relationship was also seen between *Verrucomicrobia* and high-density lipoprotein cholesterol, thus suggesting an implication of these bacteria in the observed changes in total cholesterol levels between procedures following surgery. The difference in the observed changes may be attributed to the difference in pH between RYGB and LSG. In RYGB, the distal stomach and the small intestine are excluded from the digestive transit; hence, the stomach acidity is in part avoided and the amount of hydrochloric acid in the intestine is reduced. pH is an important determinant of the distribution of major fermentation end products and it has been also demonstrated that pH may affect bacterial growth [116,117]. For instance, butyrogenic reactions occur at mildly acidic pH [118]. Accordingly, butanoate metabolism was enriched in the gut microbiome of RYGB patients, most probably mediated by *Clostridiaceae*, *Clostridium*, or *Oscillospira*, all of which are butyrate producers [119]. In a separate study, Kong et al. [120] reported an increase in microbiota richness following RYGB, mostly owing to an increase in *Proteobacteria*. In addition, an association was established between gut microbiota and gene expression in white adipose tissue. Wisnewsky et al. demonstrated that RYGB and LSG produced notable changes to gut microbiota, and that the observed differences may be associated with modifications in the digestive physiology that are caused by a surgical procedure [121]. For instance, *Firmicutes* phyla in RYGB correlated with glycated hemoglobin levels and improved trunk fat mass, whereas an increase in *Alistipes shahii* after surgery was associated with metabolic improvement.

### 4.4. Vegan and Vegetarian Diet

Vegan and vegetarian diets are gaining popularity for their reputed health protective properties, believed to be the consequence of reduced levels of inflammation and linked to their distinct gut microbiota [122]. A general pattern observed in vegan and vegetarian populations is an increase in the abundance of bacteria involved in the fermentation of dietary fiber, such as *Ruminococcus*, *Lactobacillus*, *Clostridium*, *Eubacterium rectale*, and *F. prausnitzii* [123,124]. Results of observational studies assessing the effect of vegan diet on gut microbiota showed an increase of phyla *Bacteroidetes* and a higher abundance of genus *Prevotella*, while studies yielded inconclusive results regarding the diversity and richness of gut microbiota [125]. On the other hand, interventional studies assessing the effects of plant-based diets on gut microbiota support findings of an increased abundance of bacteria involved in fiber breakdown, as well as an increase in butyrate-producing bacteria such as *Ruminococcaceae*, *Lachnospiraceae*, *Coprococcus*, *Roseburia*, *Blautia*, *Alistipes*, and *F. prausnitzii*, while also showing inconclusive results with respect to the diversity and richness of gut microbiota [126].

The observed change in the composition of the intestinal microbiota may be caused by a variety of factors, including the types of bacteria that are consumed directly through food, the substrates that are consumed, variations in duration of transition through the gastrointestinal system, the local pH, host secretion that is affected by dietary patterns, and the regulation of gene expression in host cells and/or bacteria [127]. Evidence extracted from studies which followed the *Firmicutes*-to-*Bacteroidetes* ratio are inconsistent, as we have already discussed. Nevertheless, the observed decrease in *Firmicutes* levels in favor of *Bacteroidetes* and *Bifidobacteria* might be explained as a response to an increased intake of resistant starches. A microbiome enriched in *Firmicutes* has been associated with an increased capacity for energy harvest and obesity, which could be beneficial in preventing and treating obesity [128]. In addition, De Filippo et al. confirmed a higher abundance of *Prevotella*, a genus of the *Bacteroidetes* phyla, as a response to a vegan diet [129]. Animal and plant-based food impact the abundance of *Bacteroides*, a genus well known for pro-inflammatory effects. *Clostridium clostridioforme, B. thetaiotaomicron*, and *Faecalibacterium prausnitzii*, all considered to offer protective effects, had a higher relative abundance among vegetarians/vegans compared to omnivores. On the other hand, there are inconsistent data regarding the impact of dietary patterns on the abundance of *Clostridium* cluster XIVa. Previous data showed a lower abundance of this species in vegetarians/vegans, but a recent study confirmed *Clostridium* cluster XIVa bacteria to be a major component of gut microbiota in vegetarian women [130]. Abundance of the third major enterotype, *Ruminococcus*, was associated with long-term fruit and vegetable consumption. Species from this enterotype specialize in degrading complex carbohydrates, such as resistant starch and cellulose found in plant-based foods, which results in the production of butyrate, a molecule that offers different beneficial roles to human health including anti-inflammatory properties, lower endotoxemia, and lower arterial stiffness [131,132]. *Ruminococcus* might also play a role in the conversion of animal-derived choline to trimethylamine [133]. This might explain the importance of an animal-based diet as well as a plant-based diet to *Ruminococcus* abundance.

A diet enriched with whole grains such as barley, brown rice, or a combination of the two increased microbial diversity in a study by Martinez et al., in which participants were followed for 28 weeks [134]. The results of short-term dietary interventions which increased fiber intake showed an opposite effect, showing reduced diversity. This could be explained by a hypothesis of transitory microbial “stress”, which has a mild but significant impact and causes an increase in *Enterobacteriaceae* as a result of the rapid dietary change caused by dietary intervention. Klimenko et al. found a positive association between the alpha-diversity value of microbial richness and long-term fruit and vegetable intake, as well as a negative association between alpha-diversity and BMI [135]. These results could be explained by a greater presence of butyrate-producing bacteria which can lower colonic pH, preventing the growth of pathogenic bacteria such as *Enterobacteriaceae* in individuals following a higher-fiber diet [136].

## 5. Future Directions and Conclusions

Based on the presented data, we may conclude that various dietary interventions can result in significant changes to both gut microbiota and metabolic pathways in obese individuals. It is worth noting that the effects do not simply depend on the type of intervention, but also on its duration and the presence of concomitant caloric restriction. Unfortunately, it is rather challenging to derive proper inferences, given that most of the data are not a result of sufficiently powered RCTs. Perhaps the strongest body of evidence is available for MD, but even for this, more data are needed to reach appropriate conclusions. The biggest task in future studies is to find out whether the response to any of these interventions can be predicted using the gut microbiota composition and/or metabolites, thus providing us with a feasible method of finding the appropriate dietary intervention. Currently, there are multiple obstacles preventing us from fulfilling this task. For a start, although it was demonstrated that gut microbial changes can occur rapidly, it remains elusive what duration, if any, is needed to induce a permanent change to the core microbial profile. Secondly, the composition of one’s gut microbiome seems to be more closely related to specific dietary preferences rather than the nutritional interventions, thus further impeding the adequate designing of the studies. In line with this, nutritional intervention studies are commonly burdened by adherence issues, which poses a problem, especially in the absence of reliable adherence indicators. Finally, although there is an abundance of data in this regard, a gap still exists in our understanding of the role of gut microbiota and metabolites in the pathophysiology of obesity and metabolic syndrome. For instance, we conclude about the effects of a certain diet on a certain disorder by using serum biomarkers and, in many cases, the serum biomarkers have very limited accuracy in depicting the respective disorder.

## Data Availability

Not applicable.
